# Protective Effects of Astragalin Against Acute Ultraviolet B-Induced Photodamage in HaCaT Cells and Mouse Skin

**DOI:** 10.3390/ijms27093839

**Published:** 2026-04-26

**Authors:** Pei Tang, Yan Wang, Xuanhao Huang, Jialing Tong, Lijun Feng, Dong Fan, Yuxin Ma, Shuang Wu, Cuiling Qi

**Affiliations:** Laboratory of Tumor and Immunity, School of Basic Medical Sciences, Guangdong Pharmaceutical University, Guangzhou 510006, China; tangpei@gdpu.edu.cn (P.T.); 2112255039@stu.gdpu.edu.cn (Y.W.); huangxuanhao@gdpu.edu.cn (X.H.); 2112458025@stu.gdpu.edu.cn (J.T.); 2112558034@stu.gdpu.edu.cn (L.F.); 2112355035@stu.gdpu.edu.cn (D.F.); mayuxin@gdpu.edu.cn (Y.M.); wushuang@gdpu.edu.cn (S.W.)

**Keywords:** astragalin, photodamage, UVB irradiation, oxidative stress, skin inflammation

## Abstract

Astragalin (AST), a natural flavonoid found in various plants, possesses antioxidant and anti-inflammatory properties. However, its protective efficacy against ultraviolet B (UVB)-induced cutaneous damage remains unclear. This study investigated the photoprotective effects of AST against UVB-induced photodamage using HaCaT keratinocytes and Kunming mice. In vitro, AST mitigated UVB-induced cytotoxicity and apoptosis in HaCaT cells. In vivo, topical application of AST attenuated UVB-induced erythema, epidermal hyperplasia, and collagen degradation in mouse skin. Additionally, AST reduced reactive oxygen species accumulation and enhanced antioxidant enzyme activity via activation of the Keap1/Nrf2 pathway. Furthermore, AST suppressed the expression of proinflammatory cytokines by inhibiting the TLR4/NF-κB signaling pathway. These findings demonstrate the photoprotective properties of AST and support its potential as a natural therapeutic agent for preventing UVB-induced skin damage.

## 1. Introduction

Humans are exposed to sunlight daily, and ultraviolet (UV) radiation is a major contributor to skin damage. UV radiation is classified into three wavelength ranges: UVA (315–400 nm), UVB (280–315 nm), and UVC (100–280 nm). Among these, UVB shows limited penetration and is almost entirely absorbed by keratinocytes in the epidermis [1]. Excessive exposure to UVB radiation leads to the buildup of reactive oxygen species (ROS), resulting in inflammation, oxidative stress, and cellular apoptosis. These processes contribute to acute photodamage, premature skin aging, and a higher risk of cutaneous malignancies [2].

Keratinocytes are the primary cell type in the human epidermis and are essential for maintaining the skin’s barrier, making them a central focus in studies on UV-related skin damage [3]. Research increasingly shows that oxidative stress and acute inflammation are key mechanisms underlying skin photodamage. UVB irradiation generates excess ROS that exceeds the skin’s endogenous antioxidant defenses, leading to oxidative injury [4]. ROS also activate key cellular signaling pathways and transcription factors, including Toll-like receptor 4 (TLR-4) and nuclear factor-κB (NF-κB), which enhance inflammatory cytokine production in keratinocytes, leading to further inflammation and skin damage [5]. Therefore, preventing UVB-induced damage in keratinocytes could represent a promising strategy for mitigating skin photodamage.

Flavonoids, a large class of polyphenolic secondary metabolites widely distributed in plants, have emerged as promising candidates for biomedical applications owing to their remarkable biological activities and high biosafety [6]. Accumulating evidence indicates that flavonoids possess potent antioxidant, anti-inflammatory, and cytoprotective effects, making them valuable for mitigating oxidative stress-related disorders, including skin photodamage [7]. However, the translational potential of flavonoids is often limited by low bioavailability, insufficient metabolic stability, and inefficient intracellular accumulation, all of which directly determine their actual therapeutic efficacy in target tissues [8]. A recent comprehensive review systematically highlighted that optimization of delivery systems and pharmacokinetic properties can significantly enhance the intracellular efficacy of flavonoids, particularly for cutaneous applications requiring sustained antioxidant and anti-inflammatory activity [9].

**Figure 1:**
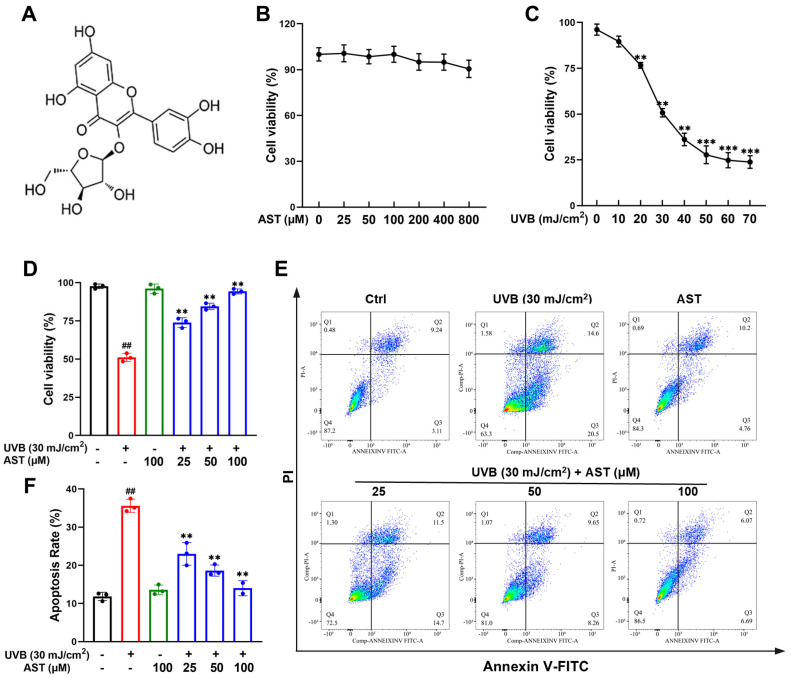
AST alleviates UVB-induced damage in HaCaT cells. (**A**) Chemical structure of AST. (**B**) Cytotoxicity of AST at various concentrations in HaCaT cells. (**C**) Effects of various UVB doses on HaCaT cell viability after 24 h. (**D**) Effects of AST on cell viability following UVB irradiation. (**E**) Apoptosis analysis by flow cytometry. (**F**) Quantification of apoptotic cells. Error bars indicate mean ± SD (n = 3). ^##^ *p* < 0.01 versus control; ** *p* < 0.01, *** *p* < 0.001 versus UVB group.

Astragalin (AST; Figure 1A), or kaempferol-3-O-β-D-glucoside, is a flavonoid present in various fruits and plants such as persimmons, cherries, and Cassia alata. AST has antioxidant, anti-allergic, and anti-inflammatory effects [10]. In previous studies, AST was found to alleviate cellular damage in a model of AlCl3/galactose-induced aging by attenuating oxidative stress, inflammation, and apoptosis [11]. It also improved the skin condition and reduced transdermal water loss in an NC/Nga mouse model of atopic dermatitis [12]. However, its potential role in protecting against UVB-induced skin damage remains unclear. Therefore, in this study, we aimed to investigate the protective effects of AST and to explore its potential as a novel strategy for enhancing skin protection.

## 2. Results

### 2.1. AST Increases the Viability of UVB-Irradiated HaCaT Cells

Figure 1A presents the chemical structure of AST. To assess its cytotoxicity, we treated HaCaT cells with increasing concentrations (0–800 µM) and measured viability using a CCK-8 assay. As illustrated in Figure 1B, no significant decrease in cell viability was observed across this concentration range. Next, the appropriate UVB dose was determined by exposing HaCaT cells to varying UVB intensities and measuring resultant cell viability. A dose of 30 mJ/cm^2^ was selected for subsequent experiments, as it induced approximately a 50% reduction in cell viability compared to that of unexposed controls (Figure 1C). The IC50 parameter is generally used as the modeling dose [13]. HaCaT cells were then pre-treated with AST prior to UVB irradiation at an intensity of 30 mJ/cm^2^. Pretreatment with AST before UVB exposure significantly improved HaCaT cell viability in a dose-dependent fashion (Figure 1D). Additionally, Annexin V-FITC/PI double staining demonstrated that UVB exposure significantly increased the proportion of Annexin V-FITC-positive cells, whereas AST pretreatment markedly suppressed UVB-induced apoptosis in HaCaT cells (Figure 1E,F). UVB exposure significantly decreased Bcl-2 expression and increased Bax and Caspase-3 levels relative to the control group, whereas AST pretreatment dose-dependently reversed these changes and restored the Bcl-2/Bax ratio toward an anti-apoptotic state (Figure S1).

Collectively, these findings indicate that AST protects HaCaT cells from UVB-induced damage.

### 2.2. AST Inhibits Oxidative Stress in UVB-Irradiated HaCaT Cells

**Figure 2:**
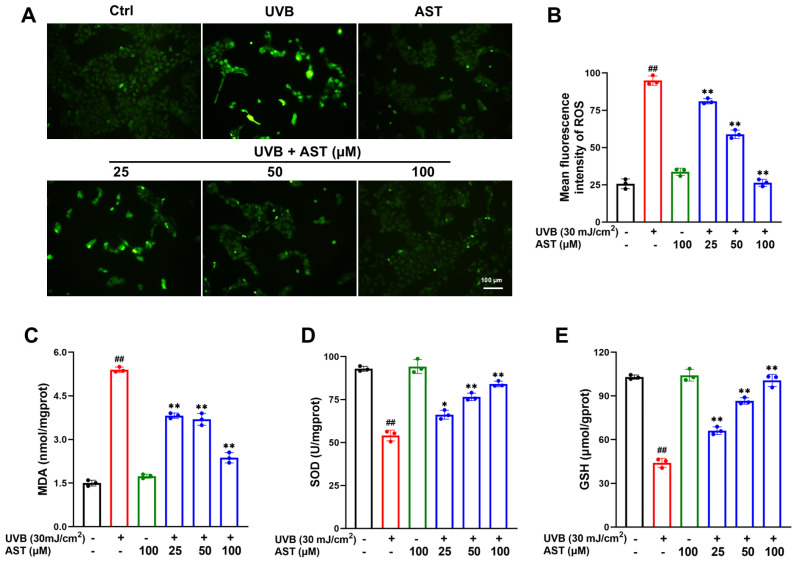
AST attenuates UVB-induced intracellular ROS generation and oxidative damage in HaCaT cells. (**A**) ROS content was determined by DCFH-DA staining. Scale bar is 100 µm. (**B**) Quantitative analysis of fluorescence intensity. Levels of (**C**) MDA, (**D**) SOD, and (**E**) GSH across different treatment groups. Error bars indicate mean ± SD (n = 3). ^##^ *p* < 0.01 versus control; * *p* < 0.05, ** *p* < 0.01 versus UVB group.

To determine whether AST could alleviate UVB-induced oxidative stress, intracellular ROS production was measured using the fluorescent probe DCFH-DA, and antioxidant enzyme activities were assessed using commercial kits. As shown in Figure 2A, UVB exposure significantly increased intracellular ROS production, whereas AST treatment dose-dependently attenuated this accumulation. Furthermore, UVB irradiation led to an increase in the MDA content and a reduction in SOD activity and GSH levels. These alterations were significantly reversed following AST treatment (Figure 2C–E). Collectively, these findings indicate that AST effectively protects against UVB-induced oxidative damage in HaCaT cells.

### 2.3. AST Alleviates UVB-Induced Inflammation in HaCaT Cells

**Figure 3:**
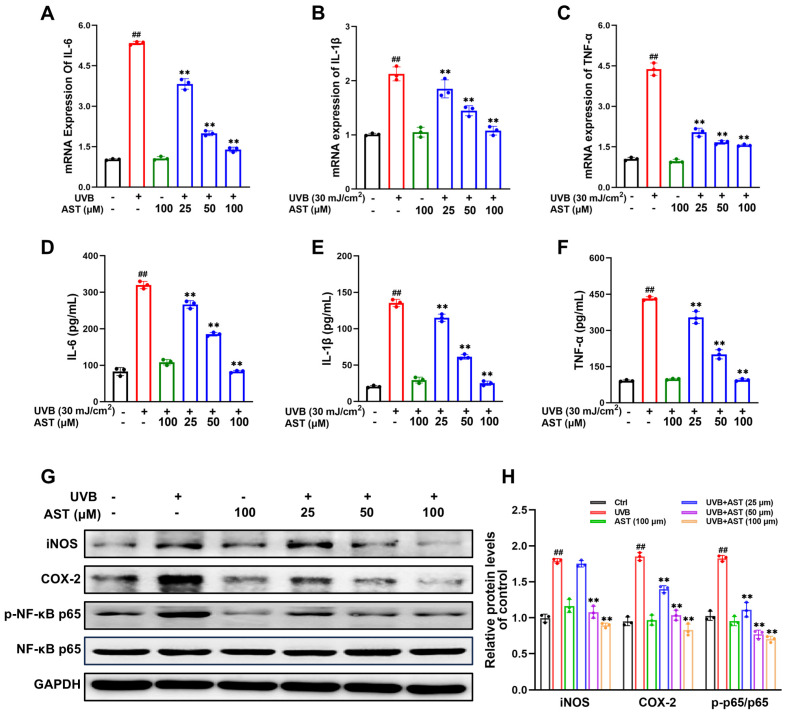
AST alleviates UVB-induced inflammatory responses in HaCaT cells. (**A**–**C**) mRNA levels for IL-6, IL-1β, and TNF-α were analyzed by RT-qPCR. (**D**–**F**) Secretion of these cytokines in the supernatant was quantified using ELISA. (**G**) Western blotting was performed to evaluate the protein expression of iNOS, COX-2, p-NF-κB p65, and NF-κB p65. (**H**) Quantification of protein expression. Error bars indicate mean ± SD (n = 3). ^##^ *p* < 0.01 versus control; ** *p* < 0.01 versus UVB group.

In HaCaT cells exposed to UVB irradiation (Figure 3A–C), mRNA expression of the proinflammatory cytokines IL-6, IL-1β, and TNF-α was significantly upregulated. These effects were attenuated in a dose-dependent manner following pretreatment with AST, and corresponding elevations in secreted protein levels were confirmed by ELISA analysis of the culture supernatant (Figure 3D–F). Furthermore, UVB exposure stimulated the protein expression of COX-2 and iNOS, both of which were suppressed by AST treatment (Figure 3G). NF-κB is a critical regulator of inflammatory responses in epidermal homeostasis. Western blot data revealed that UVB exposure significantly increased the phosphorylation of NF-κB p65 relative to controls. This phosphorylation was inhibited by AST treatment in UVB-irradiated HaCaT cells (Figure 3G). Thus, AST reduces UVB-induced inflammation in HaCaT cells.

### 2.4. Topical Application of AST Protects Against Acute UVB-Induced Skin Damage in Mice

**Figure 4:**
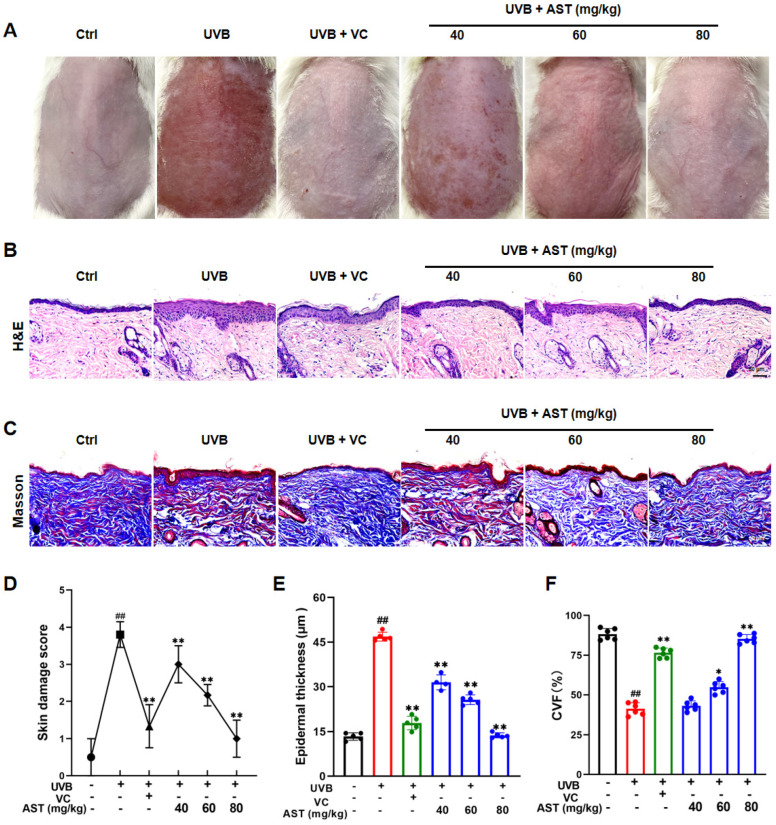
AST alleviates UVB-induced skin damage in KM mice. (**A**) Photographs of the dorsal skin post-UVB irradiation. (**B**) H&E staining and (**C**) Masson’s trichrome staining of skin sections. Scale bar is 50 μm. (**D**) Scoring of photodamage. (**E**) Measurement of epidermal thickness. (**F**) Quantitative analysis of collagen volume fraction. Error bars indicate mean ± SD (n = 6). ^##^ *p* < 0.01 versus control; * *p* < 0.05, ** *p* < 0.01 versus UVB group.

An acute UVB photodamage model was established in Kunming (KM) mice to evaluate the protective effects of AST in vivo, with vitamin C (VC, 100 mg/kg) as the positive control [14]. As shown in Figure 4A, dorsal skin exposed to UVB exhibited diffuse erythema, edema, roughness, and desquamation. Compared with the control, AST treatment reduced these symptoms in a dose-dependent manner (Figure 4A).

The skin damage scores of AST-treated KM mice significantly decreased in a concentration-dependent manner compared with control group, while the damage scores of AST (80 mg/kg)-treated mice were also lower than those of VC (100 mg/kg)-treated mice (Figure 4D).

H&E and Masson’s trichrome staining (Figure 4B,C) demonstrated that the epidermis in the control group remained thin and structurally intact, with evenly distributed collagen fibers in the dermis. In contrast, UVB exposure induced epidermal thickening, hyperkeratosis, intracellular edema, and collagen degradation.

Epidermal thickness of AST-treated KM mice was significantly reduced in a concentration-dependent manner relative to that of control mice, while epidermal thickness of AST (80 mg/kg)-treated mice was also significantly reduced compared to that of VC (100 mg/kg)-treated mice (Figure 4E). The collagen volume fraction in both the AST (80 mg/kg) and VC (100 mg/kg) groups was significantly higher than that in the control group (Figure 4F). Importantly, the skin damage scores and epidermal thickness in AST-treated mice were significantly reduced compared with those in VC-treated mice, demonstrating that AST exhibited a stronger ability to repair UVB-induced damage (Figure 4D,E).

Thus, AST exerts protective effects against UVB-induced skin photodamage in mice.

### 2.5. AST Inhibits UVB-Induced Oxidative Damage by Activating the Keap1/Nrf2 Pathway

**Figure 5:**
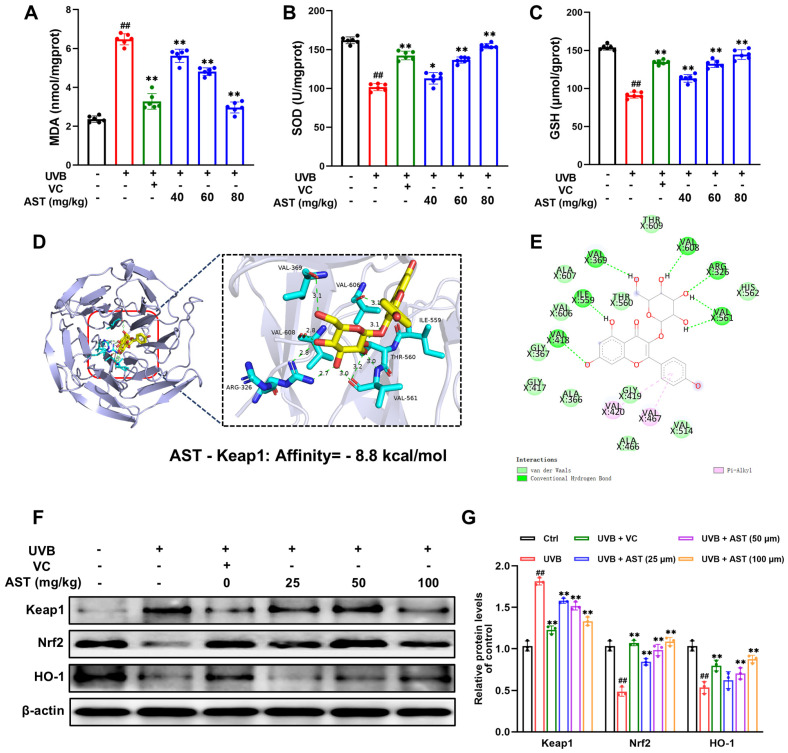
AST inhibits UVB-induced oxidative damage in mouse skin. (**A**) MDA, (**B**) SOD, and (**C**) GSH levels in skin tissues of different groups (n = 6). (**D**) Three-dimensional docking image of AST with Keap1. (**E**) Two-dimensional interaction diagram of AST and Keap1. (**F**) Representative Western blots showing the expression of Keap1, Nrf2, and HO-1. (**G**) Quantification of protein expression levels (n = 3). Error bars indicate mean ± SD. ^##^ *p* < 0.01 versus control; * *p* < 0.05, ** *p* < 0.01 versus UVB group.

We examined the activities of key antioxidant enzymes in the dorsal skin of UVB-exposed mice to investigate the antioxidant effects of AST in vivo. As depicted in Figure 5A, MDA levels were significantly elevated in the UVB group. Administration of AST or VC markedly reduced MDA accumulation relative to the UVB group. Similarly, UVB exposure resulted in marked decreases in SOD activity and GSH levels compared to the controls. These reductions were significantly reversed in the AST-treated groups. Likewise, compared with the UVB group, the VC positive control group also had partially recovered SOD and GSH levels, but its efficacy was weaker than that of high-dose AST (Figure 5B,C).

The Keap1/Nrf2 pathway plays a key role in regulating cellular antioxidant defenses and is essential in mitigating UVB-induced oxidative damage. Molecular docking analysis revealed that AST formed hydrogen bonds with Keap1 at amino acid residues VAL-369, ILE-559, VAL-418, ARG-326, VAL-561, and VAL-608, with a high affinity of −8.8 kcal/mol, indicating a stable interaction (Figure 5D,E). Subsequent analysis of skin tissues confirmed that UVB exposure downregulated Nrf2 and HO-1 protein expression while upregulating Keap1. Both AST and VC treatments normalized these parameters by suppressing Keap1 and enhancing Nrf2/HO-1 expression (Figure 5F,G).

Collectively, these findings indicate that AST alleviates UVB-induced oxidative damage by modulating the Keap1/Nrf2/HO-1 pathway and confirm a similar protective mechanism for VC.

### 2.6. AST Reduces the Inflammatory Response in UVB-Induced Skin Tissue by Inhibiting the TLR4/NF-κB Signaling Pathway

**Figure 6:**
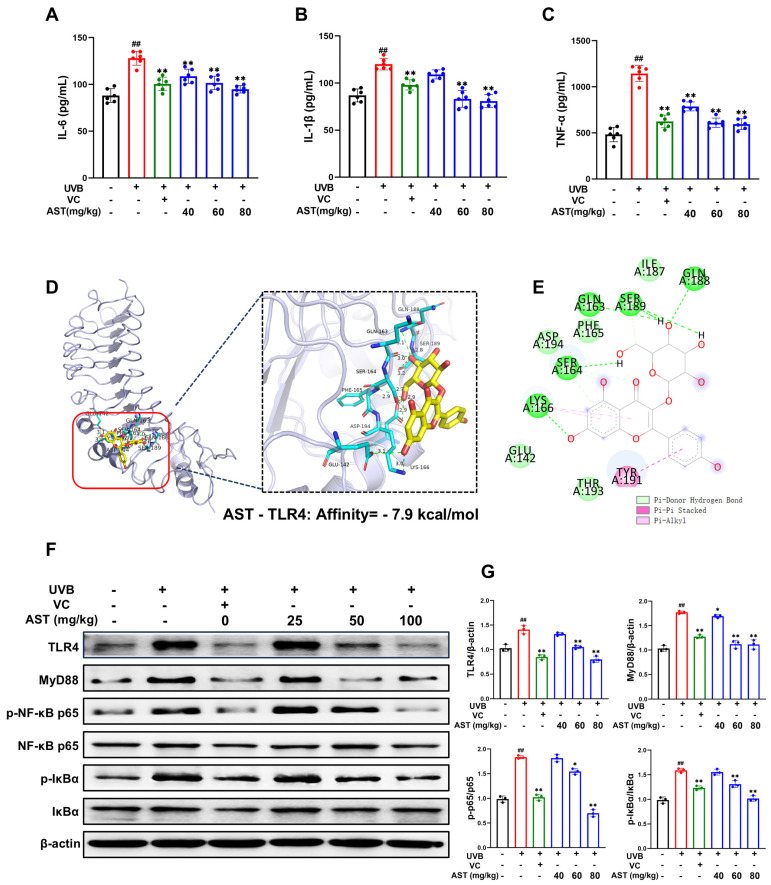
AST reduces UVB-induced acute inflammation in mouse skin. (**A**–**C**) ELISA-based quantification of IL-6, IL-1β, and TNF-α levels in skin tissue (n = 6). (**D**) Three-dimensional molecular docking image of AST and TRL4. (**E**) Two-dimensional interaction diagram of AST and TLR4. (**F**) Western blot analysis of TLR4, MyD88, p-NF-κB p65, NF-κB p65, p-IκBα, and IκBα expression in skin tissues of mice. (**G**) Quantification of protein levels (n = 3). Error bars indicate mean ± SD. ^##^ *p* < 0.01 versus control; * *p* < 0.05, ** *p* < 0.01 versus UVB group.

UVB-induced inflammation is a key contributor to skin damage. In this study, UVB exposure significantly elevated the levels of proinflammatory cytokines IL-6, IL-1β, and TNF-α in mouse dorsal skin, whereas treatment with AST or VC significantly suppressed these elevated levels (Figure 6A–C). Molecular docking analysis revealed that AST formed stable interactions with the TLR4 binding site, with a high affinity of −7.9 kcal/mol (Figure 6D,E), suggesting that TLR4 is a direct molecular target of AST.

Moreover, Western blot results demonstrated that AST treatment significantly inhibited UVB-induced upregulation of TLR4, MyD88, p-IκBα, and NF-κB p-p65 in vivo (Figure 6F,G).

These results suggest that the protective effect of AST against UVB-induced skin inflammation is, at least in part, mediated by suppression of the TLR4/NF-kB signaling pathway.

## 3. Discussion

As the body’s outermost barrier, the skin serves as its first line of defense against environmental hazards. With the progressive depletion of the atmospheric ozone layer, UV light reaching the Earth’s surface has intensified, contributing to a rise in photodamage-associated skin disorders [15]. Although conventional chemical sunscreens are widely used to reduce UV-induced damage, their application may be associated with adverse effects, including neurotoxicity, contact dermatitis, and photosensitivity [16]. This has prompted growing interest in natural compounds that can mitigate UVB-induced oxidative stress.

AST is a natural flavonoid extracted from various plant species. Its molecular structure is characterized by multiple phenolic hydroxyl groups (-OH), which are known to confer potent free radical scavenging activity [17]. Notably, astragalin is a glycosylated flavonoid, and emerging evidence indicates that glycosylation profoundly influences the physicochemical properties, membrane permeability, intracellular stability, and signaling selectivity of flavonoids [18]. Compared with their aglycone counterparts, glycosylated flavonoids often exhibit more balanced redox activity, reduced nonspecific cytotoxicity, and enhanced preferential targeting of Nrf2-mediated antioxidant signaling and NF-κB-driven inflammatory regulation. Such structural determinants may explain the relatively low toxicity and pathway-specific effects of astragalin observed in our study. AST can attenuate UVB-induced actinic keratosis and prevent the progression of squamous cell carcinoma [19]. Moreover, AST has demonstrated low cytotoxicity and minimal side effects, supporting its potential as a safe and promising photoprotective agent [20].

Excessive exposure to UVB radiation initiates a unified signaling cascade centered on ROS overproduction, which acts as the upstream trigger linking oxidative stress to inflammatory responses [21]. Recent studies have established that flavonoids protect against UVB-induced skin injury by coordinately regulating the Nrf2–NF-κB axis, wherein redox balance controlled by Nrf2 directly determines the magnitude of NF-κB-driven inflammation [22,23]. Consistent with this framework, UVB-induced ROS overproduction exceeds the skin’s endogenous antioxidant capacity, triggering oxidative stress and skin injury such as sunburn and hyperpigmentation. Previous research has demonstrated that AST reduces mitochondrial ROS generation and enhances antioxidant enzyme levels in dermal fibroblasts [24]. In line with these findings, our study shows that pretreatment with AST notably enhanced SOD and GSH activities and decreased MDA levels in both HaCaT cells and mouse dorsal skin. These effects suggest that AST effectively counteracts UVB-induced oxidative stress and lipid peroxidation.

Nrf2 is a key transcriptional regulator of antioxidant responses. Its activation and downstream targets mitigate UV-induced photooxidative damage and support tissue repair [25]. Flavonoids such as andrographolide modulate Nrf2 signaling and reduce ROS production [26]. AST is a potential modulator of the Nrf2 pathway. For example, AST alleviates cisplatin-induced renal damage by activating the Nrf2/Keap1 pathway [27]. In line with these observations, molecular docking analyses in our study revealed that AST formed hydrogen bonds with Keap1, thereby disrupting its interaction with Nrf2 and promoting the translocation and expression of HO-1. Collectively, these findings support the involvement of the Keap1/Nrf2/HO-1 signaling cascade in mediating the protective effects of AST against UVB-induced oxidative damage.

In addition to inducing oxidative damage, UVB irradiation promotes inflammation by stimulating keratinocytes to secrete proinflammatory cytokines, resulting in erythema, edema, and tissue damage [28]. As a downstream consequence of dysregulated ROS and insufficient Nrf2 activity, NF-κB is hyperactivated, leading to excessive cytokine release [29]. In our study, UVB-exposed mouse skin exhibited elevated levels of IL-6, IL-1β, and TNF-α, all of which reduced significantly following AST treatment, underscoring its anti-inflammatory effects.

NF-κB, a crucial transcription factor triggered by UVB exposure, regulates the expression of various inflammatory mediators [30]. Our results demonstrated that AST suppressed the phosphorylation of NF-κB p65 and downregulated iNOS and COX-2 in UVB-irradiated cells. These observations align with prior findings demonstrating the efficacy of AST in suppressing LPS-induced inflammatory responses in intestinal epithelial cells by reducing COX-2, iNOS, and cytokines expression [31]. TLR4, a major upstream regulator of NF-κB, contributes to UVB-induced inflammation. AST inhibits the TLR4/ NF-κB signaling pathway in various disease contexts, including lung injury, hepatic fibrosis, and skin inflammation [32]. Our findings, supporting this mechanism, revealed that UVB exposure increased TLR4/MyD88 expression and promoted NF-κB activation, whereas AST suppressed TLR4 expression, inhibited NF-κB phosphorylation, and ultimately reduced the release of inflammatory cytokines. Thus, in this study, AST effectively reversed this cascade by scavenging ROS, restoring Nrf2 activation, suppressing NF-κB phosphorylation, and ultimately reducing the release of inflammatory cytokine. Its antioxidant and anti-inflammatory properties make it a promising therapeutic agent for preventing photodamage.

From a translational perspective, the pharmacokinetic profile of AST is well-suited for skin photoprotection. Compared with aglycones (kaempferol/quercetin), which exhibit high permeability but poor metabolic stability and low bioavailability, glycosylated flavonoids such as AST possess enhanced stability and rely on SGLT1 transporters to maintain sustained intracellular concentrations [33]. Notably, lipophilic aglycones readily cross the blood–brain barrier, posing potential neurotoxicity risks, whereas AST shows minimal central nervous system exposure—an important safety advantage for topical application. For cutaneous delivery, aglycones penetrate the epidermis more rapidly, whereas AST demonstrates superior epidermal retention, thereby prolonging local efficacy [34]. Recent advances in delivery strategies (nanoemulsions, phytosomes, liposomes) effectively address the solubility and permeability limitations of AST, further optimizing its cutaneous bioavailability and translational potential for UVB-induced photodamage treatment [35].

Despite these findings, this study has some limitations. First, it focused only on acute UVB-induced skin damage, without addressing long-term effects such as chronic photodamage or photocarcinogenesis. Second, no direct evidence was provided for Nrf2 nuclear translocation and ARE-driven transcriptional activation of downstream target genes. Although the protein expression and molecular docking data support involvement of the Keap1/Nrf2 pathway, these key mechanistic events require further validation. Third, while molecular docking and protein expression analyses suggest involvement of specific signaling pathways, further mechanistic studies using gene knockdown or inhibitor models are warranted to confirm the direct targets. Lastly, the pharmacokinetics and skin absorption profile of AST were not examined, which may affect its translational feasibility in clinical applications.

## 4. Materials and Methods

### 4.1. Reagents

AST (purity > 98%; Cat. No. 111655-201503) was sourced from Yuan Ye Bio-Technology Co., Ltd. (Shanghai, China). Human immortalized keratinocytes (HaCaT cells) were acquired from the Cell Bank of the Chinese Academy of Sciences (Shanghai, China). Primary antibodies against Nrf2, Keap1, HO-1, COX-2 and p-NF-κB p65 were purchased from Abcam (Cambridge, UK). Antibodies targeting TLR4, MyD88, iNOS, and NF-κB p65 were purchased from Proteintech (Wuhan, China), whereas antibodies against IκBα, and p-IκBα, GAPDH, and β-actin were acquired from Cell Signaling Technology (Danvers, MA, USA).

### 4.2. Cell Culture, AST Pretreatment, and UVB Irradiation

HaCaT cells were maintained in a complete growth medium [Dulbecco’s Modified Eagle Medium (DMEM), containing 1% penicillin/streptomycin and 10% fetal bovine serum (all from Gibco, Grand Island, NY, USA)] at 37 °C in 5% CO_2_. AST was prepared as a 10 mM solution in PBS and stored at −20°C. The procedures for UVB irradiation and AST treatment followed previously established protocols [36]. Briefly, cells were pretreated for 24 h with or without AST, rinsed gently with PBS, and subsequently exposed to UVB (320–400 nm) at 30 mJ/cm^2^ using a UVB lamp (Philips, Hamburg, Germany). Following irradiation, cells were washed and replenished with fresh medium.

### 4.3. Cell Viability Assay

HaCaT cells were plated in 96-well culture dishes and permitted to adhere overnight. The cells were then treated with various treatment conditions, including different AST concentrations (0–800 µg/mL) and UVB exposure levels (0–70 mJ/cm^2^). After treatment, the cells were washed and incubated with 10 µL of CCK8 regent (Bimake, Houston, TX, USA) for 1 h. Absorbance was quantified at 450 nm utilizing a microplate reader (BioTek, Winooski, VT, USA).

### 4.4. Apoptosis Determination

HaCaT cells were washed with pre-cooled PBS, harvested using EDTA-free trypsin, and stained for apoptosis using an Annexin V-FITC/PI detection kit (Elabscience Biotechnology, Wuhan, China). Briefly, cells were resuspended in 100 μL of assay buffer and stained with 5 μL each of Annexin V-FITC and PI working solutions. The mixture was thoroughly vortexed and underwent a 20 min incubation. Apoptotic cell populations were then analyzed by flow cytometry (BD Pharmingen, San Diego, CA, USA), and the resulting data were processed using the FlowJo software (version 10.8.1).

### 4.5. ROS Generation

Intracellular ROS levels were measured following the manufacturer’s instructions with a ROS assay kit (Beyotime, Shanghai, China). HaCaT cells were cultivated on 24-well plates pretreated with AST for 24 h and subsequently exposed to UVB irradiation. Following treatment, cells were collected, rinsed twice with PBS, and incubated with 0.5 mL DCFH-DA (10 μM) for 25 min. Intracellular fluorescence was detected using an inverted fluorescence microscope (Olympus, Tokyo, Japan) and quantified by ImageJ software (version 1.54f).

### 4.6. Determination of SOD Activity, GSH, and MDA Levels

The concentrations of SOD, GSH, and MDA were assessed both in vitro and in vivo using corresponding commercial kits (Jiancheng Bioengineering Institute, Nanjing, China). HaCaT cells were harvested and lysed via sonication. Skin tissues were homogenized in cold PBS to yield a 10% tissue solution. The samples were processed following the manufacturer’s guidelines, encompassing steps such as sample addition, enzyme incubation, color development, reaction termination, and final absorbance measurement.

### 4.7. Detection of Inflammatory Cytokines

Pro-inflammatory cytokines, including IL-6, IL-1β, and TNF-α, were quantified in both mouse skin tissues and cell culture supernatants utilizing specific ELISA kits (Laizee Biotech, Shanghai, China).

### 4.8. Real-Time PCR Analysis

Total RNA was isolated from treated cells on ice using TRIzol reagent (Invitrogen, Carlsbad, CA, USA), and reverse-transcribed into complementary DNA (cDNA) using a commercial kit (Accurate Biology, Shanghai, China). Real-time quantitative PCR was subsequently performed on a LightCycler^®^ 2.0 system (Roche, Basel, Switzerland). All amplification primer are shown in Supplementary Table S1.

### 4.9. Animal Experiment

Male KM mice (6–8 weeks old) were obtained from the Laboratory Animal Center of Guangdong Pharmaceutical University and housed in accordance with institutional guidelines for animal experimentation. A total of 36 mice were randomly allocated into six groups (n = 6 per group): Control (no VUB), UVB model, positive control (Vitamin C, 100 mg/kg), and UVB + AST-treated groups (40, 60, and 80 mg/kg). AST and VC were both dissolved in PBS and diluted to their respective working concentrations. Dorsal hair was removed using depilatory cream 24 h before treatment. AST (40, 60, or 80 mg/kg) or VC (100 mg/kg) was topically applied on the dorsal surface of each mouse for 30 min prior to UVB exposure, while mice in the control group were treated with the corresponding volume of PBS. Except for the control group, mice were exposed to UVB (2 J/cm^2^) to cause acute UVB photodamage as previously described [37,38]. Skin photographs and tissue samples were collected the following day. The criteria for skin photodamage scoring were referenced from the reported method [39]. All procedures were approved by Guangdong Pharmaceutical University’s Experiment al Animal Ethics Committee (Approval No. Gdpulac 2023156).

### 4.10. Histological Analysis

Paraffin-embedded sections of dorsal skin (4 µm thick) were prepared through fixation in 4% paraformaldehyde, followed by dehydration, clearing, and embedding. After deparaffinization, H&E staining was used to assess epidermal thickness, whereas Masson’s trichrome staining was utilized for examining collagen deposition. All staining procedures were performed according to the manufacturer’s protocols using commercially available reagents (Solaibao Technology Co., Ltd., Beijing, China). Histopathological changes in the skin tissues were visualized under the microscope (Olympus, Tokyo, Japan), and epidermal thickness and collagen volume fraction (CVF) were calculated using ImageJ software. CVF = (Collagen area/Total image area) × 100%.

### 4.11. Western Blotting

Cells and dorsal skin tissues were lysed using a RIPA buffer (KeyGEN, Nanjing, China), and total protein concentrations were quantified via a BCA assay kit (Thermo Scientific, Waltham, MA, USA). Protein samples were then subjected to electrophoresis and transfer onto a polyvinylidene difluoride membrane. After blocking with QuickBlock™ Blocking Buffer (Beyotime, Shanghai, China), the membrane was incubated overnight at 4°C with gentle agitation using the following primary antibodies diluted in blocking solution: anti-NF-κB (p65), anti-p-NF-κB (p65), anti-Keap1, anti-Nrf2, anti-TLR4, anti-MyD88, anti-IκBα, anti-p-IκBα (all at 1:1000), and anti-GAPDH and anti-β-actin (both at 1:10,000). After thrice washing with TBST, appropriate HRP-conjugated secondary antibodies (1:2000; Cwbio, Beijing, China) were applied for 1 h. Chemiluminescent signals were detected using an ECL substrate (Millipore, MA, USA), and band intensities were analyzed using ImageJ software for quantitative densitometry.

### 4.12. Molecular Docking

Molecular docking simulations were used to predict interactions between AST and core protein targets. The three-dimensional structures of Keap1 and TLR4 were obtained from the Protein Data Bank (PDB, https://www.rcsb.org/), and the structure of AST was retrieved from the PubChem database (https://pubchem.ncbi.nlm.nih.gov/). Protein structures were prepared by adding hydrogen atoms and optimizing charges using AutoDock Tools (version 1.5.7). Binding energies were calculated using AutoDock Vina (version 1.1.2), and docking outcomes were visualized using PyMOL (version 2.6.2) and Discovery Studio (2019).

### 4.13. Statistical Analysis

All experiments were conducted in triplicate. Data are expressed as the mean ± standard deviation (SD). Statistical analyses were performed using SPSS version 26.0 (IBM Corp., Armonk, NY, USA). Comparisons between two groups were analyzed using Student’s *t*-tests, whereas one-way ANOVA was applied for multiple group comparisons. Significance was defined as a *p*-value less than 0.05.

## 5. Conclusions

In summary, this study demonstrated that AST effectively protects against UVB-induced damage in both HaCaT cells and KM mice. The protective mechanisms appear to be mediated by the suppression of ROS production and inflammatory cytokine release through inhibition of the TLR4/NF-κB signaling pathway, as well as by activation of the Keap1/Nrf2 pathway to enhance antioxidant defenses. These findings provide a theoretical basis for the potential application of AST as a preventive agent against UVB-induced skin damage.

## Data Availability

The original contributions presented in this study are included in the article and Supplementary Materials. Further inquiries can be directed to the corresponding author.

## Supplementary Materials

The following supporting information can be downloaded at: https://www.mdpi.com/article/10.3390/ijms27093839/s1.

## Abbreviations

The following abbreviations are used in this manuscript:

AST	Astragalin
UVB	Ultraviolet B radiation
ROS	Reactive Oxygen Species
Nrf2	Nuclear factor erythroid 2-related factor 2
Keap1	Kelch-like ECH-associated protein 1
HO-1	Heme oxygenase-1
TLR4	Toll-like receptor 4
NF-κB	Nuclear factor kappa-light-chain-enhancer of activated B cells
iNOS	Inducible nitric oxide synthase
COX-2	Cyclooxygenase-2
SOD	Superoxide dismutase
GSH	Glutathione
MDA	Malondialdehyde
MyD88	Myeloid differentiation primary response 88
HaCaT	Human adult low calcium temperature keratinocytes (cell line)

## References

[1] Ezekwe N., Maghfour J., Kohli I. (2022). Visible Light and the Skin. Photochem. Photobiol..

[2] Guan L.L., Lim H.W., Mohammad T.F. (2021). Sunscreens and Photoaging: A Review of Current Literature. Am. J. Clin. Dermatol..

[3] Bai F., Fan C., Lin X., Wang H.Y., Wu B., Feng C.L., Zhou R., Wu Y.W., Tang W. (2023). Hemin protects UVB-induced skin damage through inhibiting keratinocytes apoptosis and reducing neutrophil infiltration. J. Photochem. Photobiol. B.

[4] Lu W., Kong C., Cheng S., Xu X., Zhang J. (2023). Succinoglycan riclin relieves UVB-induced skin injury with anti-oxidant and anti-inflammatory properties. Int. J. Biol. Macromol..

[5] Ansary T.M., Hossain M.R., Kamiya K., Komine M., Ohtsuki M. (2021). Inflammatory Molecules Associated with Ultraviolet Radiation-Mediated Skin Aging. Int. J. Mol. Sci..

[6] Domaszewska-Szostek A., Puzianowska-Kuźnicka M., Kuryłowicz A. (2021). Flavonoids in Skin Senescence Prevention and Treatment. Int. J. Mol. Sci..

[7] Anbualakan K., Tajul Urus N.Q., Makpol S., Jamil A., Mohd Ramli E.S., Md Pauzi S.H., Muhammad N. (2022). A Scoping Review on the Effects of Carotenoids and Flavonoids on Skin Damage Due to Ultraviolet Radiation. Nutrients.

[8] Gupta M., Ahmad J., Ahamad J., Kundu S., Goel A., Mishra A. (2023). Flavonoids as promising anticancer therapeutics: Contemporary research, nanoantioxidant potential, and future scope. Phytother. Res..

[9] Rana J.N., Gul K., Mumtaz S. (2025). Isorhamnetin: Reviewing Recent Developments in Anticancer Mechanisms and Nanoformulation-Driven Delivery. Int. J. Mol. Sci..

[10] Chen J., Zhong K., Qin S., Jing Y., Liu S., Li D., Peng C. (2023). Astragalin: A food-origin flavonoid with therapeutic effect for multiple diseases. Front. Pharmacol..

[11] Hu Y., Fang X., Wang J., Ren T.T., Zhao Y.Y., Dai J.F., Qin X.Y., Lan R. (2022). Astragalin attenuates AlCl_3_/D-galactose-induced aging-like disorders by inhibiting oxidative stress and neuroinflammation. Neurotoxicology.

[12] Matsumoto M., Kotani M., Fujita A., Higa S., Kishimoto T., Suemura M., Tanaka T. (2002). Oral administration of persimmon leaf extract ameliorates skin symptoms and transepidermal water loss in atopic dermatitis model mice, NC/Nga. Br. J. Dermatol..

[13] Long Y., Wang W., Zhang Y., Du F., Zhang S., Li Z., Deng J., Li J. (2023). Photoprotective Effects of Dendrobium nobile Lindl. Polysaccharides against UVB-Induced Oxidative Stress and Apoptosis in HaCaT Cells. Int. J. Mol. Sci..

[14] Tan H., Ren H., Chai J., Zhai C., Li T., Zhou X., Lee J., Li X., Zhao Y. (2024). Protective effect of ginseng berry saponin conversion products on skin photodamage caused by UVB in vitro and in vivo. Food Res. Int..

[15] Cui B., Wang Y., Jin J., Yang Z., Guo R., Li X., Yang L., Li Z. (2022). Resveratrol Treats UVB-Induced Photoaging by Anti-MMP Expression, through Anti-Inflammatory, Antioxidant, and Antiapoptotic Properties, and Treats Photoaging by Upregulating VEGF-B Expression. Oxid. Med. Cell. Longev..

[16] Tanveer M.A., Rashid H., Tasduq S.A. (2023). Molecular basis of skin photoaging and therapeutic interventions by plant-derived natural product ingredients: A comprehensive review. Heliyon.

[17] Kim E.H., Shim Y.Y., Lee H.I., Lee S., Reaney M.J.T., Chung M.J. (2022). Astragalin and Isoquercitrin Isolated from Aster scaber Suppress LPS-Induced Neuroinflammatory Responses in Microglia and Mice. Foods.

[18] Rana J.N., Mumtaz S. (2025). Prunin: An Emerging Anticancer Flavonoid. Int. J. Mol. Sci..

[19] Li N., Zhang K., Mu X., Tian Q., Liu W., Gao T., Ma X., Zhang J. (2018). Astragalin Attenuates UVB Radiation-induced Actinic Keratosis Formation. Anti-Cancer Agents Med. Chem..

[20] Yang C.Z., Wang S.H., Zhang R.H., Lin J.H., Tian Y.H., Yang Y.Q., Liu J., Ma Y.X. (2023). Neuroprotective effect of astragalin via activating PI3K/Akt-mTOR-mediated autophagy on APP/PS1 mice. Cell Death Discov..

[21] Qu C., Liang S., Wang K., He Y., Ju W., Sun Y., Miao J. (2025). EPA-enriched lipid from Apostichopus japonicus byproducts mitigates UVB-induced oxidative stress and inflammation by gut-skin axis. Food Res. Int..

[22] Zheng S.L., Wang Y.M., Chi C.F., Wang B. (2024). Chemical Characterization of Honeysuckle Polyphenols and Their Alleviating Function on Ultraviolet B-Damaged HaCaT Cells by Modulating the Nrf2/NF-κB Signaling Pathways. Antioxidants.

[23] Sajeeda A., Bhat A.M., Gorke S., Wani I.A., Sidiqui A., Ahmed Z., Sheikh T.A. (2024). Naringenin, a flavanone constituent from Sea buckthorn pulp extract, prevents ultraviolet (UV)-B radiation-induced skin damage via alleviation of impaired mitochondrial dynamics mediated inflammation in human dermal fibroblasts and Balb/c mice models. J. Photochem. Photobiol. B.

[24] Alblihed M.A. (2020). Astragalin attenuates oxidative stress and acute inflammatory responses in carrageenan-induced paw edema in mice. Mol. Biol. Rep..

[25] Sun J.M., Liu Y.X., Tsai Y.T., Liu Y.D., Ho C.K., Wen D.S., Tsai T.Y., Zheng D.N., Gao Y., Zhang Y.F. (2025). Salvianolic acid B protects against UVB-induced HaCaT cell senescence and skin aging through NRF2 activation and ROS scavenging. J. Photochem. Photobiol. B.

[26] Kong Y.H., Xu S.P. (2020). Juglanin administration protects skin against UVB-induced injury by reducing Nrf2-dependent ROS generation. Int. J. Mol. Med..

[27] Ijaz M.U., Furqan R., Salar M.Z., Hamza A., Ashraf A., Hamdi H. (2025). Pharmacotherapeutic potentials of astragalin against cisplatin-induced renal toxicity via regulating Nrf-2/keap-1 pathway. Arch. Physiol. Biochem..

[28] Xiao T., Chen Y., Song C., Xu S., Lin S., Li M., Chen X., Gu H. (2021). Possible treatment for UVB-induced skin injury: Anti-inflammatory and cytoprotective role of metformin in UVB-irradiated keratinocytes. J. Dermatol. Sci..

[29] Shi Y., Chen J., Li S., Wu Y., Yu C., Ni L., Xiao J., Shao Z., Zhu H., Wang J. (2022). Tangeretin suppresses osteoarthritis progression via the Nrf2/NF-κB and MAPK/NF-κB signaling pathways. Phytomedicine.

[30] Li X.Q., Cai L.M., Liu J., Ma Y.L., Kong Y.H., Li H., Jiang M. (2018). Liquiritin suppresses UVB-induced skin injury through prevention of inflammation, oxidative stress and apoptosis through the TLR4/MyD88/NF-κB and MAPK/caspase signaling pathways. Int. J. Mol. Med..

[31] Ruan J., Shi Z., Cao X., Dang Z., Zhang Q., Zhang W., Wu L., Zhang Y., Wang T. (2024). Research Progress on Anti-Inflammatory Effects and Related Mechanisms of Astragalin. Int. J. Mol. Sci..

[32] Cao M.M., Guo Z., Wang J., Ma H.Y., Qin X.Y., Hu Y., Lan R. (2025). Astragalin alleviates lipopolysaccharide-induced depressive-like behavior in mice by preserving blood-brain barrier integrity and suppressing neuroinflammation. Free Radic. Biol. Med..

[33] Zhang H., Tsao R. (2016). Dietary flavonoid glycosides: Absorption, metabolism, and bioactivity. Nutrients.

[34] Granado-Serrano D., Martín M.Á., Bravo L., Goya L., Ramos S. (2022). Bioactivity and Therapeutic Potential of Kaempferol and Quercetin. Int. J. Mol. Sci..

[35] Silva M.C., Reis S., Lima J.L. (2023). Lipid-Based Delivery Systems for Flavonoids and Flavonolignans. Int. J. Nanomed..

[36] Mei M., Cai R., Yu Q., Tian R., Zhu W., Song J., Wu D. (2023). Salidroside alleviates UVB-induced skin damage by inhibiting keratinocytes pyroptosis via the AQP3/ROS/GSDMD-N signaling pathway. J. Funct. Foods.

[37] Oliveira M.M., Daré R.G., Barizão É.O., Visentainer J.V., Romagnolo M.B., Nakamura C.V., Truiti M. (2018). Photodamage attenuating potential of Nectandra hihua against UVB-induced oxidative stress in L929 fibroblasts. J. Photochem. Photobiol. B.

[38] Wang K., You X., Qu Z., Che D., Cao X. (2024). Livin is protective in UVB-induced skin photodamage by regulating keratinocyte activation and inflammatory responses. J. Cell. Mol. Med..

[39] Han B., Wang S., Zeng Y., Wang Y., Zeng X., Yang Z., Huang X., Huang S., Wang H., Gao R. (2025). Protective effects of polysaccharides from Spirulina platensis against ultraviolet light-induced skin photoaging in KM mice and damage to mouse dermal fibroblasts. J. Funct. Foods.

