# Supplementary material for: Protective Effects of Astragalin Against Acute Ultraviolet B-Induced Photodamage in HaCaT Cells and Mouse Skin
Source: Int J Mol Sci. 2026 Apr 26;27(9):3839. doi: 10.3390/ijms27093839 (PMC13164545; doi:10.3390/ijms27093839)
Supplement: Supplementary file 1 [file ijms-27-03839-s001.zip › ijms-4235711-supplementary.pdf]

Table S1. Primer sequences used for qPCR amplification.

Primer name	Primer sequence (5′→3′)
h IL-6-F	CGAGCCCACCGGGAACGAAA
h IL-6-R	GGACCGAAGGCGCTTGTGGAG
h IL-1β-F	CCAGGGACAGGATATGGAGCA
h IL-1β-R	TTCAACACGCAGGACAGGTACAG
h TNF-F	CCTCTCTAATCAGCCCTCTG
h TNF-R	GAGGACCTGGAGTAGATGAG
h GAPDH-F	GCAAAGTGGAGATTGTTGCCAT
h GAPDH-R	CCTTGACTGTGCCGTTGAATTT

Figure S1. Effect of AST on the protein expression of Bcl-2, Bax and Caspase 3 in UVB-induced photodamaged HaCaT cells.

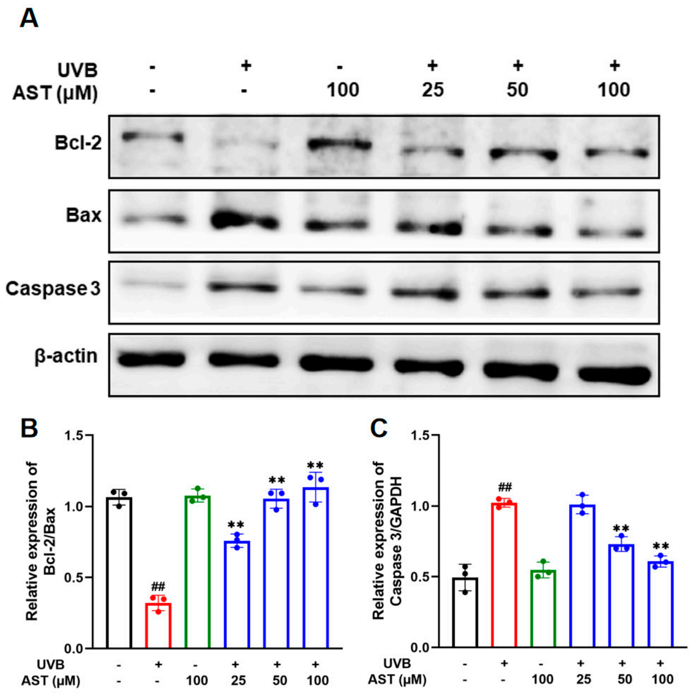

Figure S1. Effect of AST on the protein expression of Bcl-2, Bax and Caspase 3 in UVB-induced photodamaged HaCaT cells. Western blot representative images (A) and quantitative analysis of (B) Bcl-2/Bax and (C) Caspase 3. Error bars indicate mean ± SD (n = 3). ##$P < 0.01$ versus control; *$P < 0.05$, **$P < 0.01$ versus UVB group.
